# Bendlet Transform Based Adaptive Denoising Method for Microsection Images

**DOI:** 10.3390/e24070869

**Published:** 2022-06-24

**Authors:** Shuli Mei, Meng Liu, Aleksey Kudreyko, Piercarlo Cattani, Denis Baikov, Francesco Villecco

**Affiliations:** 1 College of Information and Electrical Engineering, China Agricultural University, Beijing 100083, China; meishuli@163.com (S.M.); bynd_lm@163.com (M.L.); 2 Department of Medical Physics and Informatics, Bashkir State Medical University, Lenina Str. 3, 450008 Ufa, Russia; akudreyko@bashgmu.ru; 3 Department of Computer, Control and Management Engineering, University of Rome “La Sapienza”, Via Ariosto 25, 00185 Roma, Italy; cattani.1642354@studenti.uniroma1.it; 4 Department of Surgery, Transplantology and Radiation Diagnostics, Bashkir State Medical University, Lenina Str. 3, 450008 Ufa, Russia; d-baikov@mail.ru; 5 Department of Industrial Engineering, University of Salerno, Via Giovanni Paolo II 132, 84084 Fisciano, Italy

**Keywords:** Rician noises, magnetic resonance imaging, bendlet transform, adaptive algorithm

## Abstract

Magnetic resonance imaging (MRI) plays an important role in disease diagnosis. The noise that appears in MRI images is commonly governed by a Rician distribution. The bendlets system is a second-order shearlet transform with bent elements. Thus, the bendlets system is a powerful tool with which to represent images with curve contours, such as the brain MRI images, sparsely. By means of the characteristic of bendlets, an adaptive denoising method for microsection images with Rician noise is proposed. In this method, the curve contour and texture can be identified as low-frequency components, which is not the case with other methods, such as the wavelet, shearlet, and so on. It is well known that the Rician noise belongs to a high-frequency channel, so it can be easily removed without blurring the clarity of the contour. Compared with other algorithms, such as the shearlet transform, block matching 3D, bilateral filtering, and Wiener filtering, the values of Peak Signal to Noise Ratio (PSNR) and Structural Similarity Index Measure (SSIM) obtained by the proposed method are better than those of other methods.

## 1. Introduction

Computed tomography imaging represents an indispensable source of information for medical diagnosis. It uses the Radon transform to reconstruct the distribution of cross-sectional tissue structures through X-ray imaging [1]. The pixel itself is displayed according to the mean attenuation of the tissue. Any pathological changes in the tissue(s) result in another grayscale image. However, due to the optical quantum effect, the detailed information of CT image acquisition will be covered by particle noise, resulting in a high level of noise in the reconstructed image, which seriously affects feature extraction and segmentation of the target image and multi-source image fusion [2,3,4]. MRI uses the body’s natural magnetic properties to produce detailed images from any part of the body. For imaging purposes, the hydrogen nucleus is used because of its abundance in water and fat. If any pathology occurs, the proton density changes accordingly. Noise in the MRI is caused by the thermally-driven Brownian motion of electrons within the body’s conducting tissue and within the receiving coil itself. This noise is also known as Johnson noise. In middle and high-strength fields, the patient will be a dominant noise source, unless the coil is very small. The patient noise electromotive force is caused by random radiofrequency currents circulating around a number of eddy current loops, thereby producing randomly varying magnetic fields which induce noise voltages in the RF receiving coil [5]. Thus, image denoising algorithms have important practical implications for both imaging techniques.

**Figure 1:**
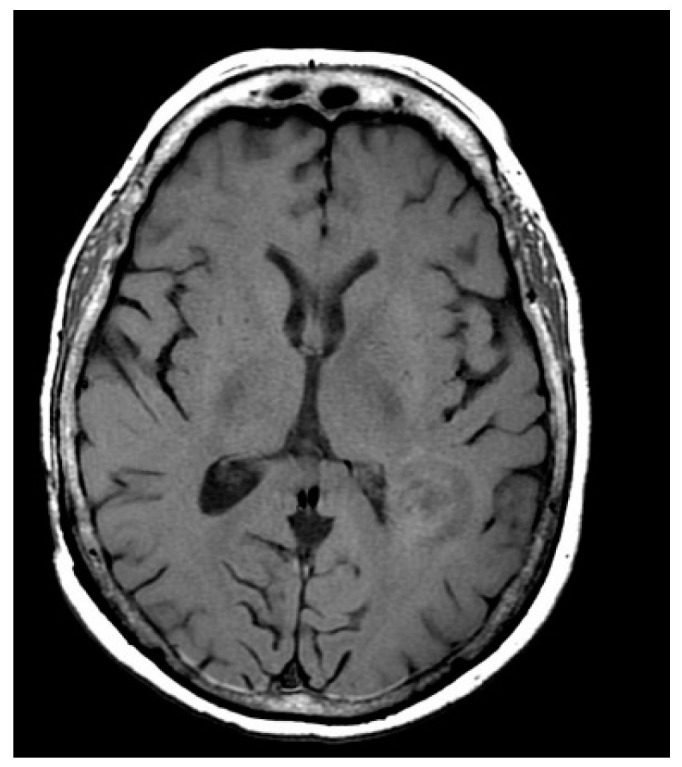
An MRI image with noise.

Typical algorithms of noise reduction often result in blurred edge, texture, and other image details. The rapid development of the convolutional neural network (CNN) provides new means for medical image processing and shows great potential [6]. However, the feature extraction capability of the CNN is limited by the dataset, hardware resources, and running time [7]. Only when the dataset is large enough is the network layer deep enough, and only the number of iterations is large enough can the network achieve effective noise reduction [8,9,10]. Deledalle et al. [11] showed that patches’ statistics followed a higher kurtosis distribution compared with the Gaussian distribution. However, patch priors are mainly limited to multivariate Gaussian or Gaussian mixture models because it is difficult to obtain estimates by them. Therefore, the prior knowledge of multi-scale mixed noise is not considered, and multiple iterations are needed to find the optimal filtering parameters for the noise image and the pre-filtered image. Pathak et al. [12] proposed a data fidelity term describing noise distribution in low-dose X-ray CT images by using a minimal likelihood logarithm. They proposed a new filter using a variational framework as the energy minimization function, which solved the data fidelity term and mixed noise problems in the sinusoidal graph data at the same time. Although this framework effectively preserves the edge of the reconstructed image, it also produces fewer gradient inversion artifacts. Xu et al. [13] proposed a fast-blind noise reduction algorithm, iQA-FBDA, which is based on image quality perception for Gaussian pulse mixed noise. It can effectively alleviate the retention of the above details and improve the calculation efficiency. Lili constructed multi-scale wavelet interpolation operators based on Shannon-Cosine wavelet [14] to achieve sparse expression of images and remove pepper and salt noise. This precise wavelet integration method based on the Shannon–Cosine wavelet operator could only eliminate Gaussian noise and salt and pepper noise of images; its elimination of Rician noise was not proven. Nevertheless, all above filtering methods do not take full advantage of the closed-loop curve profile structure appearing in images, as shown in Figure 1. In most cases, the contours and textures with large curvature are often identified as the noise in other methods. The bendlet transform can overcome this shortcoming, as it can represent the curve sparsely.

In order to show the feasibility of our method, we will focus on the details, edges, contours, and textures of several MRIs. Different tissue structures have different texture characteristics in CT/MRI, including not only typical fractal structures but also multi-layer nested annular closed contours. Muscle tissue shows an obvious regular texture with a clear texture edge. The surface layer of most tissues is a curved structure, which is smoothly curved. Each tissue structure contains rich high-frequency information. Due to this high diversity, the existing algorithms cannot accurately approximate the curve contour or curvature. The inability to identify the texture edge contour during noise reduction effectively and inevitably leads to failure to remove this high-frequency information, resulting in blurred contours [15]. The bendlets function has many excellent numerical properties, such as compact support, anisotropy, multi-scaling, shearing, and bending properties [16]. The curvature parameter can effectively identify the closed contour and remove the high-frequency noise in images. In addition, artifacts cannot be introduced into the processed images.

This study is organized as follows. Section 2 describes a preliminary review and fundamental experimental analysis. Section 3 firstly analyzes the advantages of using the bendlet transform algorithm to reduce the noise of MRIs and then compares and analyzes the noise reduction results of different algorithms to determine this study’s algorithm’s advantages. The concluding remarks are outlined in Section 4.

## 2. Preliminary Description and Methodological Framework

### 2.1. Framework of the Adaptive Noise Reduction Method Model

**Figure 2:**
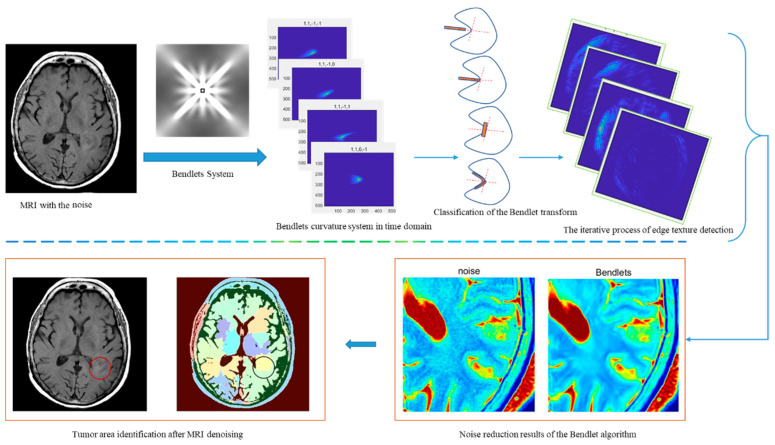
The framework of the adaptive noise reduction method model.

The overall flow of the Rician noise adaptive denoising method for microscopic images based on bendlet transform is shown in Figure 2. First, acquire a tomographic image. Then, the image description basis functions can be extended from Hilbert space to Banach space through bendlet transformation. The bendlet transform can identify the texture features of the target object in the image, especially the discontinuous texture and contour curvature caused by damage, and better approximate the texture and edge areas with rich high-frequency information. Then, the low-frequency information coefficients are reconstructed by the inverse bendlet transform to restore the denoised image, which not only preserves the orientation of the texture but also reduces the artifacts caused by shearlets in smooth regions. Finally, the method’s effectiveness is objectively demonstrated by comparing the five filtering algorithms with different image quality metrics.

### 2.2. Preliminary Remarks on Bendlets

**Figure 3:**
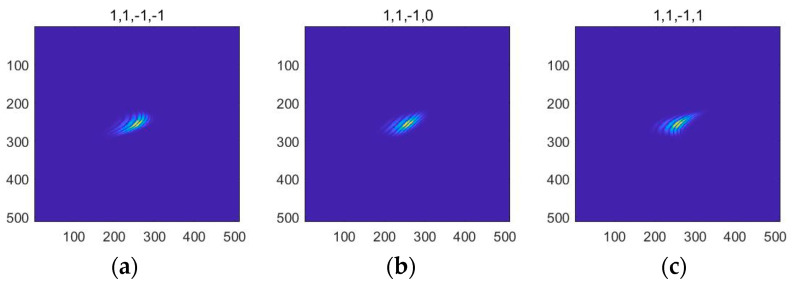
Bendlets curvature system in the time domain. (**a**) The curvature parameter is 1, indicating curvature to the right. (**b**) Curvature parameter of 0 means no curvature. (**c**) The curvature parameter is 1, which means curvature bends to the right.

**Figure 4:**
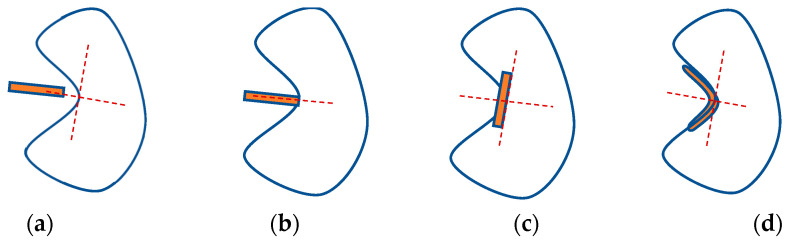
Classification of the bendlet transform: (**a**) translation parameter t is not on the edge curve. (**b**) t is on the edge curve and *s* is the boundary normal. (**c**) t is on the edge curve and s perpendicular to the normal at *t* and not corresponding to the curvature. (**d**) t is on the edge curve and *s* is perpendicular to the normal at *t* and not corresponding to the curvature.

Curvature classification in an image is a difficult task for wavelets and their generalizations, such as shearlets [17]. Although the shearlet transform can be used to detect the local direction, it cannot identify the curvature classification information of non-continuous multi-dimensional signals. In order to overcome the limitation, Lessig and the collaborators proposed a novel wavelet function named bendlet, which can describe the position of the discontinuous points, and direction and curvature of the high dimensional signal. The bendlet is developed on the basis of the shearlets, in which the curvature parameter is supplied in addition to the properties that the shearlets possess, such as the anisotropic scaling, translation, shearing, and so on. Therefore, the bendlets system is a powerful tool with which to represent the curve contour and texture. The shape of the bendlet function is shown in Figure 3.

(1) $$\phi_{a,\alpha ,s,t}(x)=a^{-\left(1+\alpha \right)/2}\phi (A^{-1}B^{-1}(x-t)),$$

where $a\in R^{+}$ is the scale parameter, α∈[0, 1], $t\in R^{2}$ is the translation, the anisotropic scale matrix is $A=\left(a,0;0,a^{\alpha }\right)$, α is the scale parameter, α=1/2 expresses the parabolic scaling, and α=1 is corresponding the isotropy scaling. B=(1,s;0,1) is the bend and shear transform matrix. The scale parameter *s* can be expressed as

(2) $$s=\sum_{m=1}^{l}r_{m}y^{m-1},\;r={\left(r_{1},r_{2},\ldots ,r_{l}\right)}^{T}\in {\mathbb{R}}^{l}$$

where l∈ℕ; x=(x,y) is the pixel position. It is easy to find that the bendlet function $\phi_{a,\alpha ,s,t}(x)$ will degenerate into the shearlet function as α=1/2; l=1 in order to obtain the edge information, which includes position, normal direction, and curvature. We should consider 4 cases, which are depicted in Figure 4.

We used circles with different radii measured in the unit square domain as input signals. When *α* = 0.5, the attenuation rate of Bendlet transform is a function of curvature. The experimental results are shown below.

**Figure 5:**
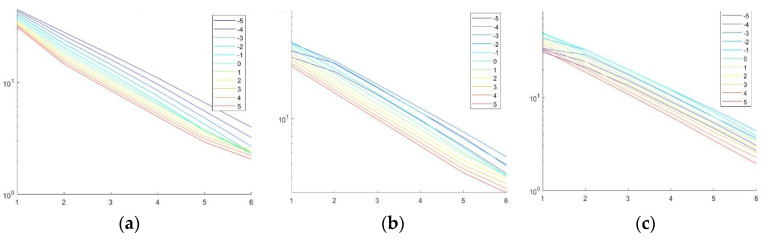
Adaptive decay rate of bendlet transform for circles with different radius. (**a**) r = 0.1. (**b**) r = 0.2. (**c**) r = 0.4.

As shown in Figure 5, it can be seen that the expected curvature sensitivity of the attenuation rate is a function of the bending parameters. For a small radius and large curvature, only the coefficients of the components with corresponding large bending decay slowly. With the increase in radius, the curvature decreases, and the smaller the bending parameters, the slower the curve decays.

**Figure 6:**
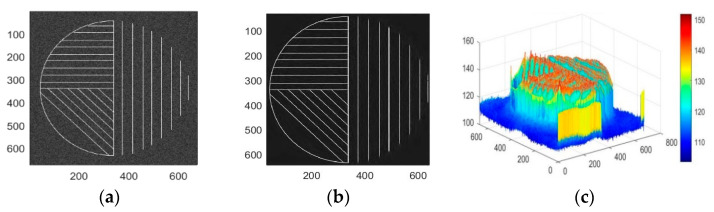
Test image of a bendlet transform. (**a**) Noisy image. (**b**) Noise-reduced image. (**c**) 3D energy image of noise reduction image.

According to the four different boundary conditions of Figure 6, the bendlets system can adaptively obtain the boundary curvature information by setting the threshold, such as translation, shearing, stretching, and bending transformation.

Figure 6a is the test image with the noise, and Figure 6b is the reconstructed image after bendlet transformation. As can be seen in Figure 6b, curvature information is added on the basis of orientation sensitivity so that the low-frequency coefficient can reflect more texture details after image decomposition, and the high-frequency coefficient can better capture the changes in image edge details. Observe the 3D energy diagram of the test image after denoising in Figure 6c. It can be seen that its energy distribution range is relatively concentrated, indicating that the texture curvature of the test image is rich and the details of the curve with smooth boundaries can also be accurately identified and processed by bendlet transformation.

## 3. Bendlet Transform for Image Denoising

### 3.1. Adaptive and Multi-Threshold Image Denoising Algorithm

**Figure 7:**
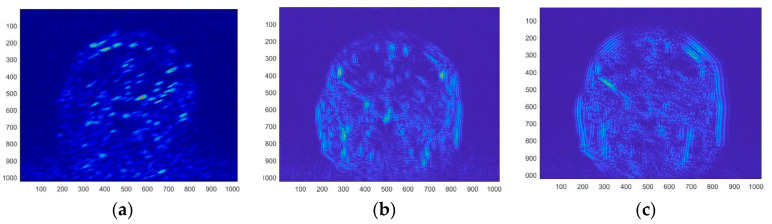
Bendlet transform of the iterative process of edge texture detection. (**a**) n = 10. (**b**) n = 50. (**c**) n = 100.

**Figure 8:**
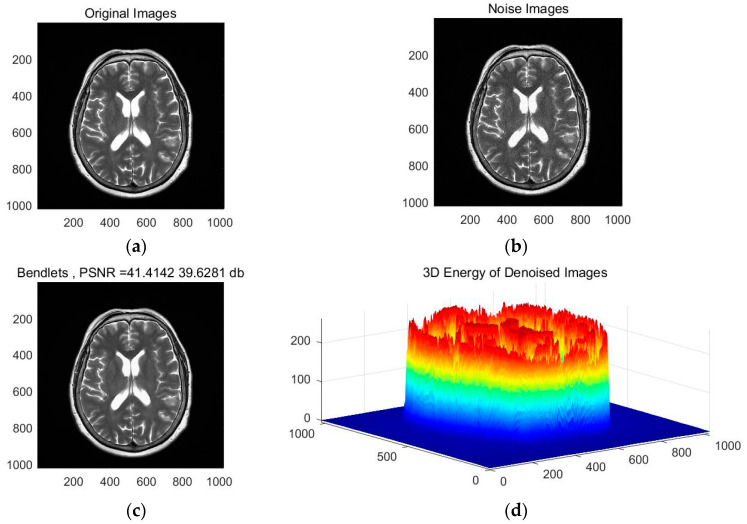
Comparison of bendlet and manual noise reduction algorithms’ results. (**a**) Original image. (**b**) Noisy image. (**c**) Noise-reduced image with Bendlets. (**d**) 3D energy image of noise reduction image.

The bendlet transform can accurately identify the details of curves with rich texture curvature and smooth boundaries for test images, indicating that features extracted from the bendlet algorithm’s band are more efficient than other image representation systems’ features. The adaptive iterative process of brain MRI denoising using the bendlet transform algorithm is shown in Figure 7, and the final results are shown in Figure 8. PSNR and SSIM were calculated to verify the effectiveness of the algorithm [18].

As shown in Figure 8, the original MIR image was de-noised by bendlets, and the PSNR and SSIM values after denoising were 41.41 dB and 0.62, respectively. Visually, the edge and texture details of the denoised image are clear. As can be seen in the 3D energy diagram in Figure 8d, irregular high-frequency mutation information was effectively removed from the image. The above analysis shows that the biological section image after the bendlet transformation and filtering not only has a clear appearance, but also lost fewer details and has strong similarity with the structure of the original image.

### 3.2. Bendlet Algorithm and Other Denoising Methods

**Figure 9:**
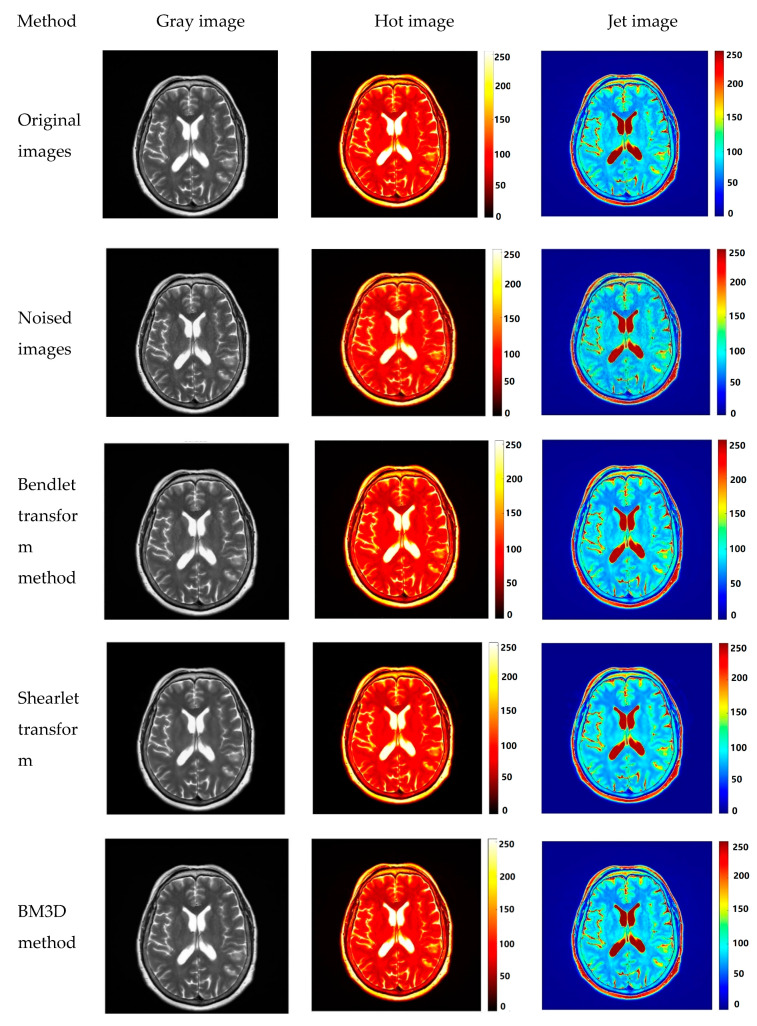

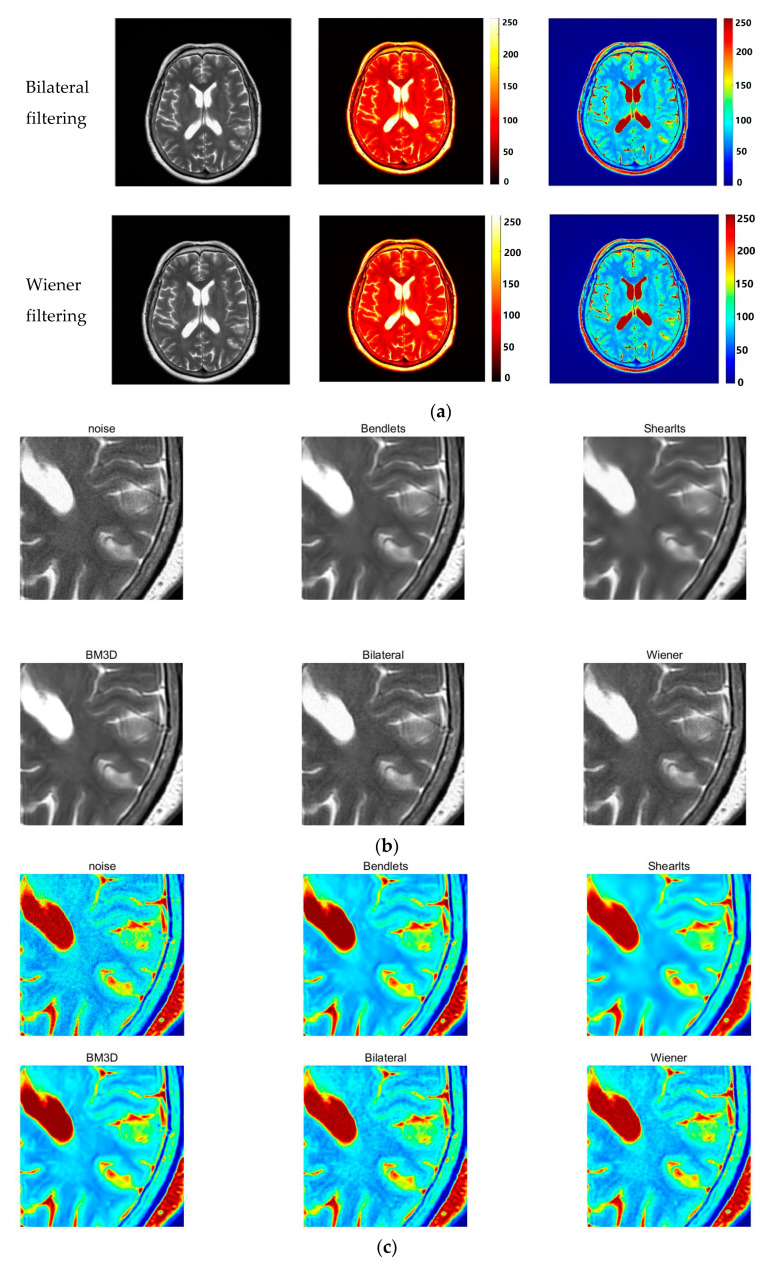
Comparison of denoising methods for MRI with noise. (**a**) Comparison of MRI noise reduction images with different methods. (**b**) Gray scale image of partially amplified MRI denoising by different algorithms. (**c**) Jet image denoising of partially amplified MRI by different algorithms.

To better reflect the advantages of the bendlet transform in MIR slice image denoising, we compare the bendlet transform with shearlets transform, bilateral filters [19], block-matching and three-dimensional filtering (BM3D) [20], the Wiener filter, and other denoising methods. Among them, the bilateral filter kernel can smoothly noise reduce while preserving edges well. BM3D is an effective combination of the characteristics of the nonlocal denoising algorithm and wavelet transform algorithm, and it is also one of the best image denoising methods at present. Hot images and Jet images can well reflect the distribution of high-frequency detail information in the image, so the degree of noise reduction in MIR can be observed. Figure 9 below shows the noise reduction results of MRI using different algorithms.

In Figure 9a, observations of hot and Jet denoising images show that bendlet, shearlt, and BM3D algorithms reduce more noise points after denoising, and gray images show that shearlet images reduce image resolution. By observing the local magnified images of Figure 9b,c, it can be known that the shearlet algorithm and BM3D can effectively identify the texture edges. The boundaries are clear, but the texture structure is different from the original image. The denoised image using bilateral filtering algorithm has fuzzy boundaries, and the denoising results of texture structure area are similar to the original image. After using Wiener filtering algorithm to reduce noise, the problem of data distortion caused by inverse transformation is prominent whether for edge recognition or texture structure reconstruction. By observing the denoising results of bendlet algorithm, compared with the original slice image, we can see that the texture and edges have high similarity to the originals.

The objective evaluation methods of PSNR, SNR, and mean squared error (MSE) use the error of the reconstructed image deviating from the original image to measure the quality of image reconstruction [21]. However, the above evaluation indicators do not consider the local visual factors of the human eye. Comparing the average pixel value of the original image and the image after noise reduction can reflect the grayscale fidelity result of the image after noise reduction to a certain extent. Variance inflation factor (VIF) measures the quality of denoised images through mutual information and expands the connection between the image and the human eye in terms of information fidelity; SSIM is based on the basis of structural information and comprehensively compares the reconstructed image and the original image from three aspects of brightness, contrast, and structure. The similarity between them makes up for the shortcoming that the VIF method is not sensitive to the structural information of the image. The calculation results of the objective evaluation indicators of noise reduction for different filtering algorithms are shown in Table 1.

The following conclusions can be drawn from the objective measurement indicators in Table 1. Take the set of image data in Table 1 as an example for analysis. It can be seen in the calculation results of the AVE value that the average pixel value of the MRI after noise reduction by the bendlet algorithm is 47.9395, and the gray average value closest to the original image is 47.9490 by the bendlets algorithm. This shows that the bendlet algorithm has the least influence on the gray value of the image before and after denoising.

PSNR is based on the error between the corresponding pixels, that is, based on the error-sensitive image quality evaluation index. The PSNR values of the five filtering algorithms in the table are 44.85, 36.46, 39.67, 35.84, and 35.74 db; and the SNR values are 34.91, 26.46, 32.73, 25.90, and 25.80, respectively. This indicates that the amounts of error in reconstructed images from different filtering algorithms compared to the original image increased in this order: bendlet, BM3D, bilateral filtering, shearlet transform, and median filtering. The calculation results of MES values show the accuracies of these algorithms, which are consistent with the PSNR values. The minimum MES value of the bendlet algorithm is 2.12, indicating that the reconstructed image has the strongest correlation with the original image. The computation time (*t*) was found in seconds using MATLAB 2020a. As can be seen in Table 1, the BM3D method had the longest running time. Bilateral and Wiener methods ran faster; however, they performed poorly in both subjective and objective evaluations. This method takes longer to compute than bendlets and shearlets, but produced better visual features. Therefore, the computation time of the proposed method is acceptable. The SSIM value and VIF value also verified the objectivity of the appearance. The SSMI values of the five algorithms are all over 0.99. This shows that the images before and after denoising have strong structural similarity, but it cannot highlight which algorithm is the best. After the curvature parameter was increased in the bendlet algorithm, the texture with rich curvature information in the MRI imaging and the nested annular closed contour in the smooth edge region were accurately approximated, and the curvatures of the edge contour and texture were also accurately classified. Therefore, the bendlet transform is suitable for texture-preserving noise reduction.

**Figure 10:**
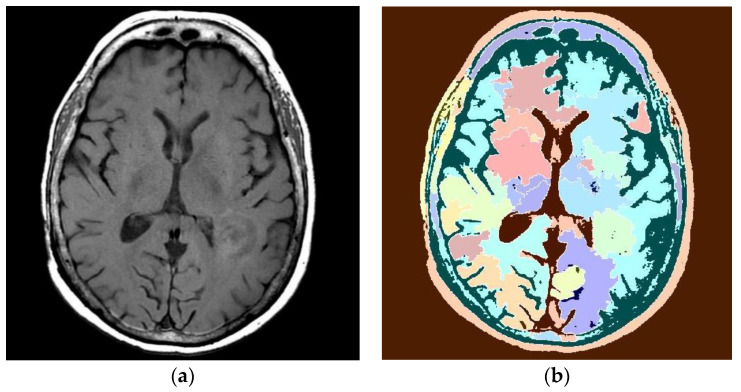
MRI of a tumor. (**a**) Original MRI. (**b**) Area recognition with original magnetic resonance image.

MRI can distinguish white matter from gray matter, so it can diagnose cerebral aneurysms and tumors, head trauma, and other soft tissue injuries and lesions. In MRIs, the locations of gliomas are nearly impossible to find because they invade nearly all locations in the brain in various shapes, sizes, and heterogeneous growth patterns. Gliomas appear very similar to other diseases in the MRIs (stroke or inflammation, etc.), and glioma cells are tangled with surrounding tissue, resulting in glioma images with diffuse borders, etc. [22]. In order to better identify the glial tumor areas in MRI images, this study mainly used bendlet transform to make the images of glioma disease in the brain better retain the definition of texture of edges after denoising, and improved the efficiency of the identification of gliomas in the brain. When MRI is obtained, many high-frequency noises will be generated to cover the texture and contour information of the lesion region [23], as shown in Figure 10a. Therefore, in the process of image fusion, the textured structure is prone to being incorrectly classified in the image without denoising processing, which makes the display of lesion edges not ideal, as shown in Figure 10b.

**Figure 11:**
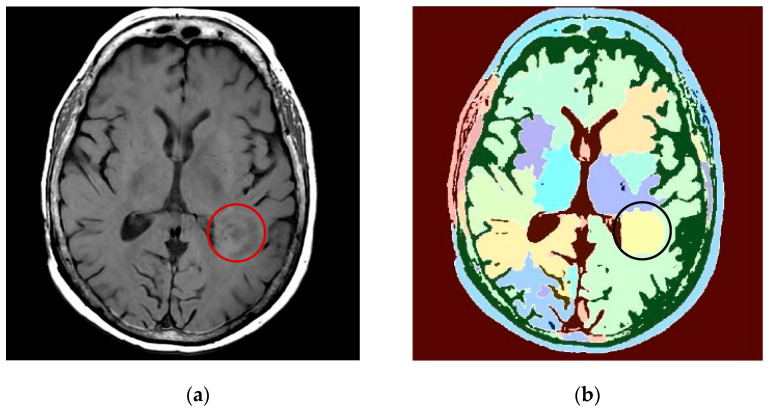
MRI of tumor lesion denoising. (**a**) MRI of tumor lesion denoising; (**b**) area recognition after MRI denoising.

The image in Figure 10b fails to meet the requirement of high-precision marking of a target area. The results after the original MIR image is denoised by changes in bendlets are shown in Figure 11.

MRIs denoised by bendlets can show soft tissue structures more accurately than original MRIs, and tumor boundary markers are clearer in areas where the original lesions are not clearly or accurately displayed, as shown in Figure 11. By comparing Figure 10b and Figure 11b, it can be seen that after denoising by bendlets, the misjudgment of classification is reduced and the accuracy of tumor area identification is improved. The results show that this method can display lesions more clearly, and is helpful for classifying various structures in MRI more accurately, so as to improve the efficiency of CT/MRI fusion and improve the accuracy of lesion target contouring.

## 4. Discussion

Based on the theory of shearlet transform and its inverse transform, we applied the bendlet algorithm based on the second-order shearlet transform to reduce the noise in view of the shortcomings of the traditional transform domain denoising algorithm, and realized the texture-preserving denoising of magnetic resonance images [24]. The bendlet transform can identify the texture characteristics of the target object in the image, especially the discontinuous texture and contour curvature caused by damage. The filtering effect is superior to that of the traditional algorithm, and it produces a clearer image. Multiple objective evaluation indexes, such as PSNR, SSIM, MSE, SNR, VIF, and time, show the superiority of our method over the traditional wavelet domain denoising algorithm and the shearlet transform denoising algorithm.

## 5. Conclusions

As a second-order shearlet system, the bendlets system can be employed to represent an object with a curve contour sparsely. The edges and texture in the images are no longer viewed as the high-frequency components in the bendlets system, which is quite different from the common wavelets system. Meanwhile, the noise is still viewed as a high-frequency component, as it is not in the form of continuous curves. Therefore, we can distinguish the noises from the edges, textures, and contours appearing in the MRI images by means of bendlet transform. This is the reason why the proposed method can remove the noise in MRI images, and the textures can be preserved perfectly. The results of the numerical experiments also illustrate this point. Compared with the other five kinds of filtering algorithm—the shearlet transform, bilateral filtering, BM3D, median filtering, and Wiener filtering—the proposed algorithm has significant advantages and can effectively remove the noise from the MRI images. The presented images were obtained from the people suspected of having space-occupying lesions. However, we decided to not discuss the diagnoses because the main aim of this paper was to show the computational performance of bendlets in medical image analysis.

## Figures and Tables

**Table 1:** Noise reduction index results for different filtering algorithms.

Filtering Algorithm	SSIM	PSNR	SNR	MSE	AVE	VIF	TIME
**Bendlets**	0.9998	44.8570	34.9154	2.1251	47.9395	0.7655	8.281
	0.9998	39.4094	29.7139	7.4497	49.4498	0.5962	8.968
	0.9997	45.3965	34.5704	6.5352	44.6905	0.5802	8.615
	0.9988	46.7255	36.1813	5.2032	40.914	0.6999	8.096
	0.9997	44.2511	34.4531	8.8191	51.932	0.7475	8.918
**Shearlets**	0.9981	36.4608	26.5192	14.6894	46.9211	0.4348	8.852
	0.9990	38.5649	28.8694	9.0487	48.9580	0.5199	7.875
	0.9987	38.786	27.960	8.5997	44.3568	0.4583	7.342
	0.9990	39.5702	29.0260	7.1789	40.2439	0.4647	8.521
	0.9987	37.8569	28.0589	10.651	51.7194	0.4723	8.334
**BM3D**	0.9995	39.6745	32.733	7.8692	48.0949	0.7414	31.955
	0.9995	38.5649	28.8694	9.0487	48.958	0.5199	34.357
	0.9993	39.9782	29.1522	6.8769	44.686	0.5502	44.051
	0.9996	40.9681	30.4238	4.3821	40.9864	0.6302	36.264
	0.9993	38.6766	28.8786	7.4433	52.0019	0.5047	33.384
**Bilateral**	0.9983	35.8376	25.8961	16.9559	47.8985	0.5273	0.377
	0.9984	39.3879	29.6924	7.4867	49.5787	0.616	0.376
	0.9983	39.3889	28.5629	7.485	44.6782	0.5003	0.368
	0.9987	42.0895	31.5453	4.0191	40.9828	0.6139	0.367
	0.9982	38.8100	29.0120	8.5522	51.9804	0.5931	0.372
**Wiener**	0.9980	35.7438	25.8023	17.326	47.9710	0.6026	0.260
	0.9980	35.4473	25.7518	18.5501	49.5007	0.6028	0.429
	0.9974	35.6611	24.8350	17.6593	44.7101	0.6258	0.303
	0.9977	35.4446	24.9003	18.5619	40.9344	0.5786	0.330
	0.9976	35.2431	25.4451	19.4434	51.9718	0.6268	0.287

## Data Availability

Not applicable.
